# Desflurane is risk factor for postoperative delirium in older patients’ independent from intraoperative burst suppression duration

**DOI:** 10.3389/fnagi.2023.1067268

**Published:** 2023-02-01

**Authors:** Susanne Koch, Benjamin Blankertz, Victoria Windmann, Claudia Spies, Finn M. Radtke, Vera Röhr

**Affiliations:** ^1^Department of Anaesthesiology and Operative Intensive Care Medicine, Charité – Universitätsmedizin Berlin, Berlin, Germany; ^2^Neurotechnology Group, Technische Universität Berlin, Berlin, Germany; ^3^Department of Anesthesia, Hospital of Nykobing, University of Southern Denmark, Odense, Denmark

**Keywords:** burst suppression EEG, intraoperative EEG monitoring, desflurane anesthesia, propofol anesthesia, elderly, postoperative delirium

## Abstract

**Background:**

Postoperative Delirium (POD) is the most frequent neurocognitive complication after general anesthesia in older patients. The development of POD is associated with prolonged periods of burst suppression activity in the intraoperative electroencephalogram (EEG). The risk to present burst suppression activity depends not only on the age of the patient but is also more frequent during propofol anesthesia as compared to inhalative anesthesia. The aim of our study is to determine, if the risk to develop POD differs depending on the anesthetic agent given and if this correlates with a longer duration of intraoperative burst suppression.

**Methods:**

In this secondary analysis of the SuDoCo trail [ISRCTN 36437985] 1277 patients, older than 60 years undergoing general anesthesia were included. We preprocessed and analyzed the raw EEG files from each patient and evaluated the intraoperative burst suppression duration. In a logistic regression analysis, we assessed the impact of burst suppression duration and anesthetic agent used for maintenance on the risk to develop POD.

**Results:**

18.7% of patients developed POD. Burst suppression duration was prolonged in POD patients (POD 27.5 min ± 21.3 min vs. NoPOD 21.4 ± 16.2 min, *p* < 0.001), for each minute of prolonged intraoperative burst suppression activity the risk to develop POD increased by 1.1% (OR 1.011, CI 95% 1.000–1.022, *p* =  0.046). Burst suppression duration was prolonged under propofol anesthesia as compared to sevoflurane and desflurane anesthesia (propofol 32.5 ± 20.3 min, sevoflurane 17.1 ± 12.6 min and desflurane 20.1 ± 16.0 min, *p* < 0.001). However, patients receiving desflurane anesthesia had a 1.8fold higher risk to develop POD, as compared to propofol anesthesia (OR 1.766, CI 95% 1.049–2.974, *p* =  0.032).

**Conclusion:**

We found a significantly increased risk to develop POD after desflurane anesthesia in older patients, even though burst suppression duration was shorter under desflurane anesthesia as compared to propofol anesthesia. Our finding might help to explain some discrepancies in studies analyzing the impact of burst suppression duration and EEG-guided anesthesia on the risk to develop POD.

## Highlights

- **Question:** Does propofol, with its higher tendency to trigger intraoperative burst suppression activity, trigger POD in older patients at a higher rate than sevoflurane or desflurane?
- **Findings:** Desflurane is associated with a higher risk to develop POD in older patients as compared to propofol or sevoflurane, even though propofol shows prolonged intraoperative burst suppression activity.
- **Meaning:** In older patients desflurane anesthesia maintenance should be used with caution, since it is associated with a higher risk to develop POD as compared to propofol or sevoflurane anesthesia maintenance.

## Introduction

Postoperative Delirium (POD) is the most frequent neurocognitive complication after general anesthesia in older patients, being associated with a higher mortality and increased morbidity (Aldecoa et al., 2017). POD is a multifactorial syndrome being related to preexisting risk factors as an age above 60 years, preoperative lower cognitive abilities, the presence of comorbidities, alcohol-related disorders and precipitating risk factors as prolonged preoperative fluid fasting, abdominal and cardiothoracic surgeries, surgery duration, intraoperative bleeding, the anticholinergic load of drug given and prolonged periods of too deep anesthesia (Chan et al., 2013; Radtke et al., 2013; Soehle et al., 2015; Fritz et al., 2016; Aldecoa et al., 2017). Periods of too deep anesthesia can be identified with intraoperative electroencephalographic (EEG) recording, were burst suppression activity is the typical signature (Brown et al., 2010; Purdon et al., 2015b). Burst suppression activity in general is an isoelectric line with intermittened bursts of alpha oscillations, indicating a highly reduced cerebral metabolism (Brown et al., 2010).

Here we like to assess the impact of different anesthetic agents on the risk to develop POD and if this is correlated to the intraoperative duration of burst suppression activity. The risk to develop intraoperative burst suppression activity is related to the dosage of the anesthetic agents given. But importantly it has been shown that the risk to present intraoperative burst suppression activity also increases with the age of the patient and shows differences depending on which anesthetic agent was given (Purdon et al., 2015a,b). Propofol has a higher tendency to induce burst suppression activity as compared to sevoflurane anesthesia (Purdon et al., 2015a). But there seems to be no EEG-slowing effect when comparing desflurane with sevoflurane (Rehberg et al., 1999).

Based on the knowledge of these former studies, we expected that older patients receiving a total intravenous anesthesia (TIVA) with propofol have (1) a higher risk to present intraoperative burst suppression activity, showing a prolonged intraoperative burst suppression duration and an increased burst suppression ratio, when compared to patients undergoing general anesthesia with either sevoflurane or desflurane. Additionally, (2) we expected to see a higher incidence of POD in older patients receiving a TIVA with propofol compared to volatile anesthetics such as sevoflurane or desflurane.

Hence, the aim of our study was first to analyze the incidence of POD in older patients in relation to the given anesthetic agent – propofol, sevoflurane or desflurane – and second to evaluate the burst suppression activity if patients who received either propofol, sevoflurane or desflurane for maintenance of general anesthesia. Finally, we wanted to know if the development of burst suppression activity in the intraoperative EEG with respect to the different anesthetic agents given intraoperatively is related to the development of POD.

## Materials and methods

The initial single center SuDoCo study was conducted as a randomized controlled trail at the Charité-Universitätsmedizin Berlin, Germany, Department of Anaesthesiology and operative intensive care medicine between March 2009 and August 2010. The ethical commission at the Charité approved the study (EA1/242/08) and all patients gave written informed consent. Data privacy and security regulations were followed and the study was registered under ISRCTN 36437985. In this secondary analysis of the single-center SuDoCo trail, we analyzed the initially 1,277 included patients. The inclusion criteria for the SuDoCo study were an age older than 60 years, the conduction of a surgery planned to last at least 60 min and the need for general anesthesia. Pre-operatively the Mini-Mental State Examination (MMSE) was performed, and patients with a MMSE score < 24 or patients who had a history of neurologic deficits were excluded (Radtke et al., 2013).

### Patients’ data assessment

Characteristics of the patients, intraoperative and postoperative data were assessed during the initial study period at the Charité from 2009 until 2010. The preoperative patient characteristics (age, American Society of Anesthesiologists’ physical status (ASA status), sex, body mass index (BMI) and Mini-Mental State Examination (MMSE)) were included in our analysis.

All patients received general anesthesia according to the standard operation procedures (SOPs) of the Charité-Universitätsmedizin Berlin (Kox and Spies, 2005). Anesthesia was induced with bolus application of thiopental, propofol, or etomidate in combination with fentanyl or remifentanil, followed by neuromuscular block to facilitate tracheal intubation. For anesthesia maintenance, patients received either total intravenous anesthesia with propofol, or inhalational anesthesia with sevoflurane or desflurane. The anesthetic agent given was chosen by the anesthesiologist in charge and was not controlled by the regime of the SuDoCo study.

POD was assessed twice daily by trained study personnel from the day of surgery until postoperative day seven by using the Diagnostic and Statistical Manual of Mental Disorders (DSM IV).

Intraoperative EEG recording was performed using the BIS monitor (Covidien, Boulder, CO, United States). EEG electrodes (electrode position Fp1, Fp2, F7 and F8) were placed in the forehead of the patient before induction of anesthesia. Bispectral Index (BIS) values indicate depth of anesthesia ranging from 0 (isoelectric line in EEG) to 100 (fully awake). BIS values between 40 and 60 indicate adequate depth of anesthesia.

### EEG data analysis

Each recorded EEG file was separately preprocessed. Periods of artifacts were excluded. Such artifacts included high amplitude artifacts, based on the 99% quantile of the data amplitude, and frequency artifacts below 0.5 Hz or above 50 Hz activity. EEG artifacts were mostly seen during anesthesia induction, caused by intubation and correct placement of the patient for surgery. After the artefact removal, the data was re-referenced by the mean. Then we determined intraoperative signatures of burst suppression activity by calculating a burst suppression probability for each time point. This probability is calculated as the ratio of time points within a second surrounding the given point that have an amplitude value below the 60% quantile of the data amplitudes. For each amplitude artifact that was removed in the first step, the second around the artifact was removed after this stage. If the probability surpassed 80% the point was marked as a suppression point. Using this as a basis, we calculated the overall burst suppression duration (min) per EEG file. The burst suppression ratio was calculated by using the fraction of burst suppression activity and the artifact-free EEG duration. All data analysis was performed with self-written scripts written in the programming languages Python and Julia using standard toolboxes and packages (PosDefManifold and PosDefManifoldML).

### Statistics

Statistical analyses were performed with SPSS, Version 26 (Copyright SPSS, Inc., Chicago, IL 60606, United States). Patients were divided into a POD and NoPOD group, according to the DSM IV results. Data are expressed as mean with standard deviation, median with 95% CI or as frequencies (%). Values were considered significant if *p* < 0.05.

Significant differences in patient characteristics were calculated by using for continuous data either the student *t*-test for age and BMI or the Mann–Whitney U-Test for MMSE, surgery duration, Burst suppression duration and burst suppression ratio. Categorical data were assessed by either the Fisher’s exact test (sex and Midazolam given) or the Pearson chi-square test (ASA status, surgical specialty, outcome, and anesthetic agent given). Differences between the anesthetic agent groups were calculated by using the Kruskal-Wallis test and the Pearson chi-square test. We also assessed the outcome parameters (days on the ward, days on ICU) and in-hospital mortality within the different anesthetic agent groups with the Kruskal-Wallis test and the Pearson chi-square test.

In a binominal logistic regression, we analyzed the independent impact of burst suppression duration and anesthetic agent used for maintenance on the risk to develop POD. We adjusted the logistic regression analysis for all predescribed POD risk factors that significantly correlated with POD in our study group.

## Results

After analyzing and preprocessing the raw electroencephalographic data files (EEG), we had to exclude 110 data files because of missing EEG raw data files or the presence of too many artifacts in the raw data files. We excluded all EEG files, where we could not at least analyze 20 min of clean raw EEG. Additionally, from 9 patients the anesthetic agent used for anesthesia maintenance was not given. Finally, we could include 1,058 patients in our analysis.

### Patients’ characteristics for POD and NoPOD

198 (18.7%) patients developed POD and 860 (81.3%) did not (NoPOD). Patients’ characteristics are given in Table 1 (detailed information see Supplementary material). POD patients had significantly prolonged burst suppression duration (POD 27.5 ± 21.3 min vs. NoPOD 21.4 ± 16.2 min, *p* < 0.001) and received more frequently desflurane for anesthesia maintenance and less frequently sevoflurane or propofol (desflurane 50.5%, sevoflurane 30.3%, propofol 19.2%, *p* < 0.001). Average and minimum BIS level of sedation did not differ between POD and NoPOD patients.

**Table 1:** Patients characteristics for patients with postoperative delirium (POD) and without (NoPOD).

	All patients (*n* = 1,058)	NoPOD (*n* = 860)	POD (*n* = 198)	Value of *p*
Age (years)	69.7 ± 6.3	69.2 ± 6.1	72.1 ± 6.5	0.000
Sex male/female (%)	54 / 46	54 / 46	53.5 / 46.5	n.s.
BMI	27.1 ± 5.0	27.3 ± 5.2	26.4 ± 4.7	0.020
ASA status (%)				0.000
1	3.1	3.8	0	
2	49.1	51.4	39.4	
3	45.8	43.4	56.6	
4	1.9	1.4	4.0	
MMSE preoperative	28.9 ± 1.4	29.0 ± 1.3	28.6 ± 1.6	0.000
Midazolam premedication yes/no (%)	5/95	5/95	7/93	n.s.
Surgical specialty (%)				0.001
General surgery	49.1	46.1	62.4	
Orthopedics	29.7	31.4	22.3	
Urology	8.1	8.1	8.1	
Gynecology	10.6	11.8	5.6	
Other	2.5	2.7	1.5	
Surgery duration (min)	170 ± 101	157 ± 92	235 ± 117	0.000
Anesthetic agents maintenance (%)				0.000
Propofol	27.8	29.8	19.2	
Sevoflurane	35.1	36.2	30.3	
Desflurane	37.1	34.1	50.5	
Depth of anesthesia				
Average BIS (0–100)	40.6 ± 7.3	40.6 ± 7.1	40.1 ± 7.8	n.s.
Minimum BIS (0–100)	32.1 ± 13.6	32.2 ± 13.4	31.2 ± 14.7	n.s.
Burst suppression duration (min)	22.5 ± 17.7	21.4 ± 16.2	27.5 ± 21.3	0.000
Burst suppression ratio	0.19 ± 0.1	0.19 ± 0.1	0.2 ± 0.11	n.s.
Outcome/discharge (%)				0.000
Home	88.9	92.5	73.6	
Other hospital	8.9	6.5	18.8	
Died	2.2	1.0	7.6	

POD patients were significant older, had lower BMI scores, lower MMSE scores, elevated ASA scores, prolonged surgery duration, prolonged burst supprestion duration and more frequently a desflurane anaesthesia. We found no significant differences in sex, premedication with midazolam, depth of anesthesia index assessed with the bispectral index (BIS) in average or minimum levels and burst suppression ratio. Sex, ASA status and anaesthetic agents used were compared by Chi-Square test. Continuious data were compared by independent *t*-test.

In spearman correlation analyses we found a high correlation of POD with age, ASA status, MMSE score, surgery duration, surgery specialization, burst suppression duration and anesthetic agent given for maintenance (Supplementary material).

### Patients’ characteristics for propofol, sevoflurane and desflurane anesthesia

**Figure 1:**
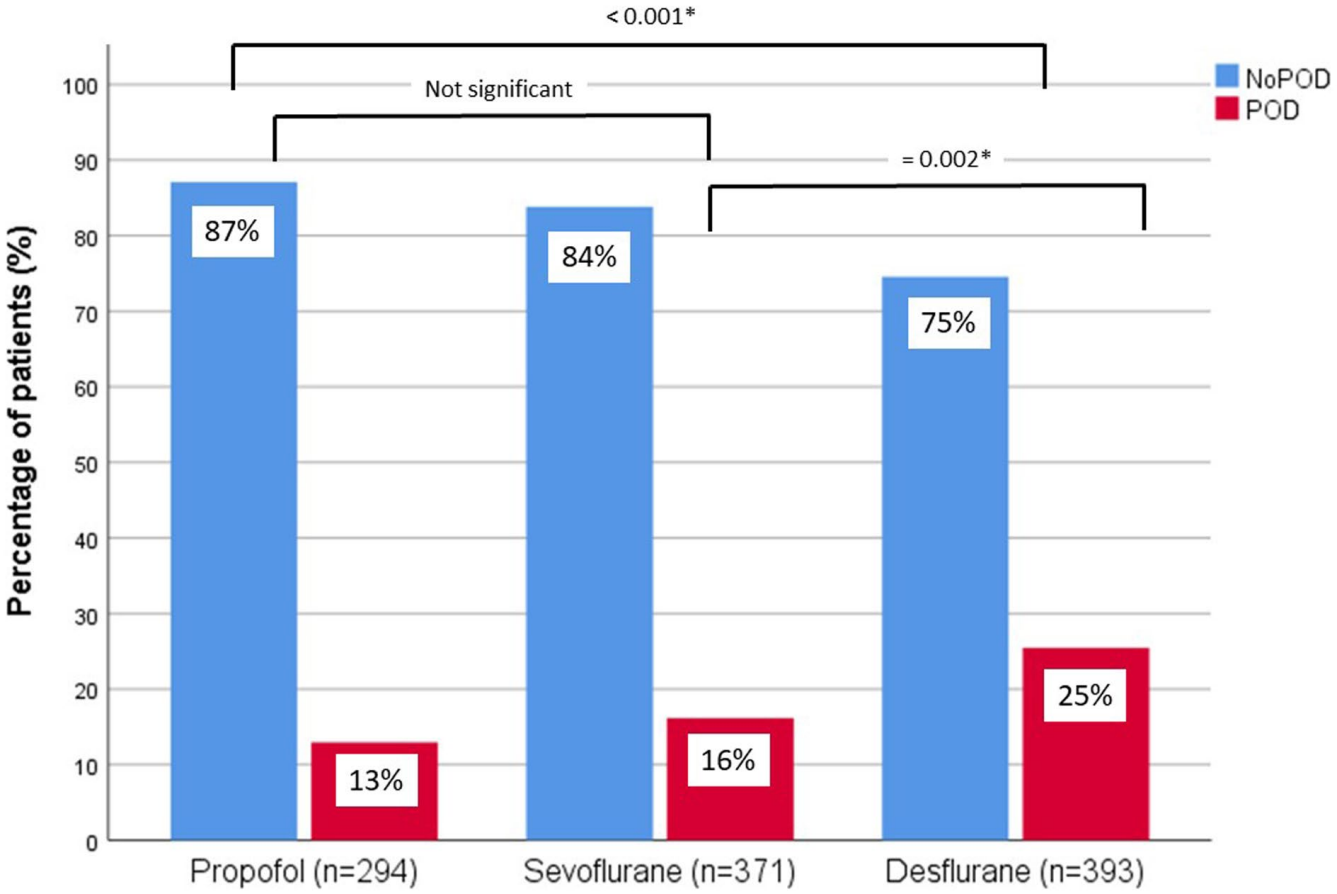
Incidence of POD and NoPOD for each anesthetic agent (propofol, sevoflurane, desflurane) in percentage of patients (%). Differences were calculated by Chi-Square test.

When comparing the patients’ characteristics between patients who received either propofol, sevoflurane or desflurane for anesthesia maintenance, we found differences in sex, BMI, surgery duration and surgical specialty. But we found no differences in age, ASA status, preoperative MMSE score and average and minimum BIS level of sedation between the patients’ groups receiving either propofol, sevoflurane or desflurane (Table 2; Supplementary material). However, we found a higher incidence of POD in the desflurane group (25.4%) compared to propofol (12.9%) or sevoflurane group (16.2%) (Table 2; Figure 1).

**Table 2:** Outcome parameter for the anesthetic agent given throughout anesthesia maintenance.

	All Patients (*n* = 1,058)	Propofol (*n* = 294)	Sevoflurane (*n* = 371)	Desflurane (*n* = 393)	Value of *p*
Burst suppression duration (min)*	22.6 ± 17.4	32.5 ± 20.3	17.1 ± 12.6	20.2 ± 16.0	0.000
Burst suppression ratio*	0.19 ± 0.1	0.25 ± 0.08	0.16 ± 0.09	0.18 ± 0.12	0.000
Depth of anesthesia					
Average BIS (0–100)	40.6 ± 7.3	40.3 ± 7.2	40.8 ± 7.4	40.8 ± 7.2	0.649
Minimum BIS (0–100)	32.1 ± 13.6	36.3 ± 6.8	36.6 ± 6.9	36.7 ± 6.9	0.633
POD yes/no (%)*	18.7/81.3	12.9/87.1	16.2/83.8	25.4/74.6	0.000
Days on the ward*	13.8 ± 11.8	13 ± 13.3	13.0 ± 10.0	15.2 ± 12.0	0.003
Days on ICU	1.5 ± 7.3	1.3 ± 7.1	1.1 ± 6.0	2.0 ± 8.6	0.527
In-hospital mortality (%)	2.2	0.3	2.2	3.6	0.001

POD and In-hospital mortality were compared by Chi-Square test. Continuious data were compared by Kruska Wallis test. *indicates *p* < 0.05.

As expected, burst suppression duration was prolonged under propofol anesthesia (32.5 ± 20.3 min) and shorter under sevoflurane (17.1 ± 12.6 min) and desflurane (20.2 ± 16.0 min) anesthesia and burst suppression ratio was highest under propofol anesthesia (0.25 ± 0.08) as compared to sevoflurane (0.16 ± 0.09) and desflurane (0.18 ± 0.12) (Table 2).

### Anesthetic agent, burst suppression activity and the impact on POD

In our binominal logistic regression analysis (including anesthetic agents given for maintenance (propofol vs. sevoflurane and propofol vs. desflurane), burst suppression duration (min), age (years), ASA status (I, II, III, IV), MMSE score (25 to 30), surgery duration (min) and surgery specialty (general surgery, orthopedics, urology, gynecology, other)) we found a significant association with POD for age, preoperative ASA score, preoperative MMSE score, surgery duration desflurane versus propofol and burst suppression duration (Table 3). Overall, we found that each minute of prolonged intraoperative burst suppression activity the risk to develop POD increased by 1.1% (OR 1.011, CI 95% 1.000–1.022, *p* = 0.046) (Table 3). Importantly, in our binominal logistic regression analysis, we found that patients had a 1.8-fold risk to develop POD after desflurane anesthesia, compared to propofol anesthesia (OR 1.766, CI 95% 1.049–2.974, *p* = 0.032), but there was no difference between propofol and sevoflurane anesthesia.

**Table 3:** Binominal logistic regression analyzing the impact on postoperative delirium (POD), including the anesthetic agent given (propofol vs. sevoflurane and propofol vs. desflurane), burst suppression duration (min), age (years), ASA status (I, II, III, IV), MMSE score (25–30), surgery duration (min), and surgery specialty (general surgery, orthopedics, urology, gynecology, other).

	Exp (B)	95% Confidence interval	Value of *p*
Sevoflurane vs. propofol	1.316	0.768–2.258	0.318
Desflurane vs. propofol*	1.766	1.049–2.974	0.032
Burst suppression duration (min)*	1.011	1.000–1.022	0.046
Age (years)*	1.084	1.053–1.115	0.000
Pre-operative ASA score*	1.574	1.146–2.163	0.005
Pre-operative MMSE score*	0.830	0.738–0.932	0.002
Surgery duration (min)*	1.000	1.000–1.000	0.000
Surgery specialty	0.852	0.711–1.022	0.085

*indicates a significant impact.

**Figure 2:**
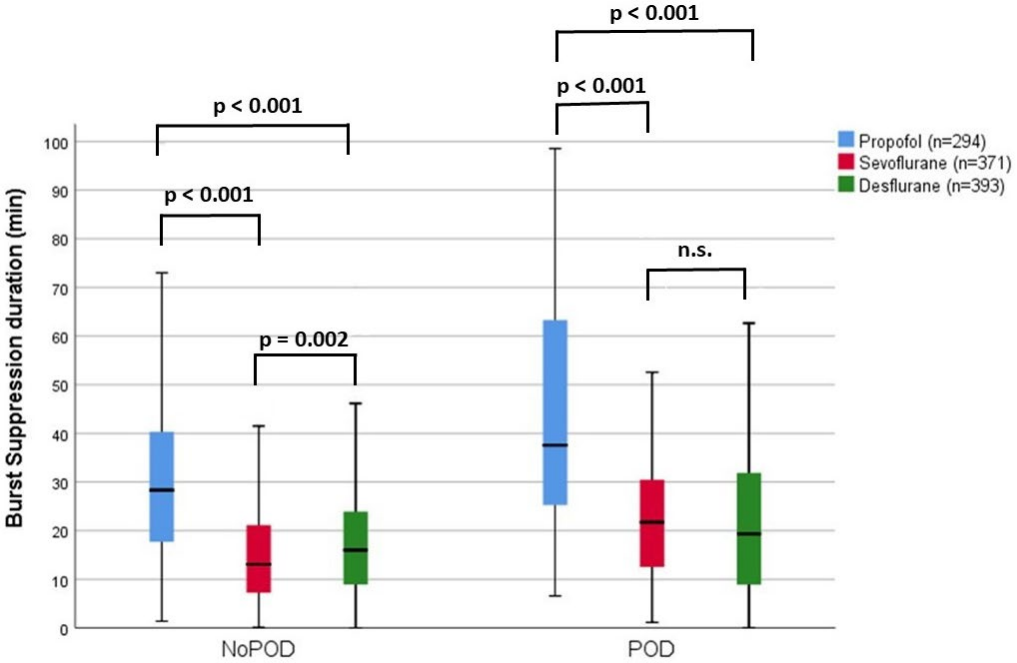
Intraoperative burst suppression duration (min) within the NoPOD and POD group within for different anesthetic agents given during anesthesia maintenance. Burst suppression duration were within the propofol group in NoPOD patients: 30.8 + 18.9 min, range 1.4 to 132.8 min and POD patients: 44.1 + 25 min, range 6.6 to 98.5 min; within the sevoflurane group in NoPOD patients: 15.8 + 11.5 min, range 0.1 to 64.1 min and POD patients: 23.9 + 15.4 min, range 1.1 to 80.7 min; within the desflurane group NoPOD patients: 19.1 + 14.2 min, range 0 to 73.6 min and POD patients: 23.3 + 20.0 min, range 0 to 133 min.

However, the risk to develop POD related to duration of burst suppression differed significantly depending to the anesthetic agent used. POD patients receiving propofol anesthesia had significantly longer burst suppression activity as compared to sevoflurane or desflurane anesthesia (Burst suppression duration of POD patients: propofol: 44.1 + 25.0 min; sevoflurane: 23.9 + 15.4 min; desflurane: 23.5 + 20.0 min; *p* = 0.025; Figure 2; Supplementary material). Additionally, patients after desflurane anesthesia stayed longer in hospital and had a higher in-hospital mortality (Table 2; Supplementary material).

## Discussion

We found a significantly increased risk to develop POD in older patients after desflurane anesthesia compared to propofol anesthesia or sevoflurane anesthesia. Interestingly this finding was independent of intraoperative burst suppression duration. Even though in general prolonged burst suppression duration was associated with a higher risk to develop POD and burst suppression duration was longest under propofol anesthesia and shortest under desflurane anesthesia, the risk to trigger POD in relation to burst suppression duration was higher under desflurane anesthesia.

Propofol seems to reduce the risk to develop POD in older patients when compared to desflurane general anesthesia, even though inducing prolonged burst suppression activity.

POD is the most frequent brain dysfunction seen after anesthesia procedures, most frequently occurring in elderly patients, where prolonged periods of too deep anesthesia are a known risk factor. It is today common sense, that EEG guidance of depth of anesthesia can reduce the risk to develop POD in elderly patients (Chan et al., 2013; Radtke et al., 2013; Whitlock et al., 2014; Punjasawadwong et al., 2018; Chan et al., 2020; Sumner et al., 2022) by avoiding too deep anesthesia (Evered et al., 2021) and prolonged periods of burst suppression (Soehle et al., 2015; Fritz et al., 2016; Xu et al., 2021). However, in recent years some randomised control trials failed to prove the advantage of EEG guidance (Zhou et al., 2018; Wildes et al., 2019; Tang et al., 2020; Wang et al., 2022). Hence, a more detailed EEG data analysis may help to improve the advantage of intraoperative EEG guidance.

Burst suppression activity in the brain presents a clear reduction of brain metabolism (Brown et al., 2010), which is discussed to be the pathophysiological reason for the higher risk to develop POD in older patients. Since it was shown that propofol has a higher ability to induce intraoperative burst suppression activity compared to sevoflurane (Purdon et al., 2015a), we would have expected a higher risk to develop POD in patients receiving propofol anesthesia. Surprisingly, we found the contrary in our study group. Patients who received propofol showed a POD incidence of 13%, after sevoflurane general anesthesia the POD rate was 16% and in patients who received a desflurane anesthesia the POD incidence was highest with 25%.

On the other hand, our data are in line with the study from Purdon et al. (2015a) showing that propofol has a higher ability to trigger burst suppression activity compared to sevoflurane. We could expand these findings by showing that desflurane also has a lower risk to trigger intraoperative burst suppression activity compared to propofol anesthesia. Importantly, we found, that the incidence of POD in relation to intraoperative burst suppression duration significantly differs between propofol and inhalative anesthetic agents. 10% of elderly patients develop POD, when presenting ~12 min of burst suppression activity under desflurane anesthesia, compared to 19 min of burst suppression activity under sevoflurane anesthesia and more than 30 min of intraoperative burst suppression under propofol anesthesia. These results indicate that a simple correlation between intraoperative burst suppression duration and the risk to develop POD in elderly patients could fail, when not including the anesthetic agent given in the analysis.

In 2011, the first study comparing the risk of developing POD between propofol and desflurane anesthesia was conducted (Royse et al., 2011). The propofol group had a slightly lower incidence of POD (7.9%) compared with the desflurane group (13.2%), but this was not significant. POD assessment was not the primary endpoint (Royse et al., 2011). In another study from 2017, 100 patients receiving either desflurane or propofol anesthesia were assessed for risk of developing POD (Tanaka et al., 2017). However, in this study, only one patient in the propofol group developed POD and no patient in the desflurane group. However, the POD surveys took place only on the first and second postoperative days, which must be considered insufficient from today’s perspective (Aldecoa et al., 2017). On the other hand, one patient in the desflurane group developed a “confused state,” but this was not designated as POD. Both studies were funded by Baxter. A more recent, larger randomized clinical trial (n = 684) compared propofol with volatile anesthesia (desflurane and sevoflurane) and the effects on the development of POD (Jiang et al., 2022). In the volatile anesthetics group, 18.7% developed POD, compared with 22.4% in the propofol group. On the other hand, this study did not distinguish between sevoflurane and desflurane. Since the significantly higher greenhouse effect of desflurane compared with sevoflurane has been discussed in recent years, it could be that the proportion of sevoflurane in the volatile group is higher than that of desflurane (Koch et al., 2022). Because we also found no difference between propofol and sevoflurane in inducing POD, this would be consistent with our data.

In the studies by Royse et al. (2011) and Jiang et al. (2022), the patients were also younger compared to our patients cohort (Royse: ~62 years; Jiang: ~54 years), which may also have contributed to the different results. For example, one might speculate that older patients have a lower ability to respond to the cerebral changes induced by desflurane administration than younger patients do.

The different anesthetic agents induce distinct intraoperative EEG signatures (Purdon et al., 2015b). Propofol shows a high, coherent alpha and slow-delta band power, whereas sevoflurane and desflurane presents additionally a theta coherent signature (Akeju et al., 2014; Purdon et al., 2015b). The pathophysiological basis behind this finding is not clear. But as propofol is a primary GABA_A_ agonist, this is the reason for it’s higher tendency to induce burst suppression activity. In contrast sevoflurane and desflurane also show GABAergic activation as well as a blockade of 2-pore potassium channels, HCN channels and they block the glutamate release by binding to NMDA receptors (Hemmings et al., 2005). Desflurane has a major inhibitory effect on voltage-gated potassium channels, whereas propofol and sevoflurane show only a lower inhibitory effect here. Propofol and sevoflurane have a major trigger effect on Glycine receptors, which is not seen under desflurane (Alkire et al., 2008). These distinct receptor affinities of the different anesthetic agents are most likely the underlying causes for their different EEG signatures (Purdon et al., 2015b) and their different ability to induce burst suppression activity (Purdon et al., 2015a). Since in preschool children, elevated neuronal excitability is related to the occurrence of emergence delirium (Martin et al., 2014; Koch et al., 2018), but not the presence of burst suppression activity (Faulk et al., 2010; Frederick et al., 2016; Koch et al., 2019), it is obvious, that there are different pathophysiological neuronal states being related to delirious symptoms. Moreover, it has been shown in molecular studies that volatile anesthetics can mediate inflammatory responses or may show neurotoxic effects (Jiang and Jiang, 2015; Yuki and Eckenhoff, 2016). Hence, one can speculate that the different receptor affinities as well as the induced neurotoxic and inflammatory effects on the cellular level of desflurane may be the underlying cause to trigger POD to a higher extent than propofol or sevoflurane do.

## Limitations

Our study is a secondary analysis and the questions we raised here were not considered the primary endpoint of the study. Moreover, the sub-groups of patients receiving either propofol, sevoflurane or desflurane differed concerning sex, BMI, surgery duration and surgery specialty, which might have biased our results. Nevertheless, since we adjusted our final analysis for the known risk factors for POD (as age, ASA status, MMSE score, and surgery duration (min) and surgery specialty) we think that our results are reliable. The patients in our study were older than 65 years; hence, our results cannot be extended to younger patients. We did not include the drugs administered for induction of anesthesia in our model. Since in most cases the first 15 min of EEG data recording could not be evaluated due to artifacts, we assume that the effect of the anesthetic administered for induction of anesthesia had already worn off by the time we could start the EEG data evaluation and thus this had no influence on the results of our evaluation.

## Conclusion

We show that propofol induces prolonged burst suppression activity in elderly patients as compared to desflurane or sevoflurane; however, this was not associated with an increased risk to develop POD. In contrast, desflurane was associated with a higher incidence of POD in our elderly cohort, independent from intraoperative burst suppression activity.

Our findings were unexpected but therefore might help to explain some discrepancies in studies analyzing the impact of burst suppression duration or the advantage of EEG neuromonitoring on the risk to develop POD. In the future, the impact of anesthetic agents on POD should be more respected and the recommendation to administer desflurane in older patients should be questioned.

## Data availability statement

The data analyzed in this study is subject to the following licenses/restrictions: Due to the nature of this research, participants of the original study did not agree for their data to be shared publicly, so supporting data is not available. Requests to access these datasets should be directed to SK, susanne.koch@charite.de. Request to access the data analysis code should be directed to VR, v.roehr@tu-berlin.de.

## Ethics statement

The studies involving human participants were reviewed and approved by Ethical commission at the Charité, Universitätsmedizin Berlin – Campus Virchow Klinikum, Augustenburger Platz 1, 13,533 Berlin. The patients/participants provided their written informed consent to participate in this study.

## Author contributions

SK conceived and designed the study, analyzed the data, wrote and revised the manuscript, and supervised the overall study. BB supervised data analysis and revised the manuscript. VW analyzed the data and revised the manuscript. CS conceived and designed the study, helped to interpret the data, and revised the manuscript. FMR conceived and designed the study, performed the experiments, helped to interpret the data, and revised the manuscript. VR performed the experiments, analyzed the data, and revised the manuscript. All authors contributed to the article and approved the submitted version.

## Funding

The research leading to these results was supported by Charité – Universitätsmedizin Berlin. The initial study has received funding from Aspect Medical Systems, now Medtronic. SK was funded by the Deutsche Forschungsgemeinschaft (DFG, German Research Foundation) – Project number KO 4249/3-1). VR acknowledges the financial support from the Research Training Group (RTG 2433) DAEDALUS (Differential Equation- and Data-driven Models in Life Sciences and Fluid Dynamics) funded by Deutsche Forschungsgemeinschaft (DFG, German Research Foundation). We acknowledge support from the German Research Foundation and the Open Access Publication Fund of TU Berlin.

## Conflict of interest

SK is an inventor on patents, sold to Medtronic. She reports a grant during the conduct of the study by the German Research Society. CS is an inventor on patents, she reports grants during the conduct of a study from European Commission, from Aridis Pharmaceutical Inc., B. Braun Melsung, Drägerwerk AG & Co. KGaA, German Research Society, German Aerospace Center, Einstein Foundation Berlin, European Society of Anesthesiology, Federal Joint committee, and Inner University grants. Grants promoting Science and Education from WHOCC, Baxter Deutschland GmbH, Cytosorbents Europe GmbH, Edwars Lifesciences Germany GmbH, Fresenius Medical Care, Grünenthal GmbH, Masimo Europe Ltd. Phizer Pharma PFE GmbH. Personal fees from Georg Thieme Verlag, Dr. F. Köhler Chemie GmbH, Sintetica GmbH, European commission, Stifterverband für die deutsche Wissenschaft e.V. /Philips, Stiftung Charite, AGUETTANT Deutschland GmbH, AbbVie Deutschland GmbH & Co. KG, Amomed Pharma GmbH, Touch Health, Copra System GmbH, Correvio GmbH, Max-Planck-Gesellschaft zur Förderung der Wissenschaft e.V., Deutsche Gesellschaft für Anästhesiologie & Intensivmedizin (DGAI), Medtronic, Philips Electronics Nederland BV, BMG and BMBF.

The remaining authors declare that the research was conducted in the absence of any commercial or financial relationships that could be construed as a potential conflict of interest.

## Publisher’s note

All claims expressed in this article are solely those of the authors and do not necessarily represent those of their affiliated organizations, or those of the publisher, the editors and the reviewers. Any product that may be evaluated in this article, or claim that may be made by its manufacturer, is not guaranteed or endorsed by the publisher.
