# Supplementary material for: Desflurane is risk factor for postoperative delirium in older patients’ independent from intraoperative burst suppression duration
Source: Front Aging Neurosci. 2023 Feb 1;15:1067268. doi: 10.3389/fnagi.2023.1067268 (PMC9929347; doi:10.3389/fnagi.2023.1067268)
Supplement: Supplementary file 1 [file Data_Sheet_1.pdf]

	All Patients (n=1,058)	Propofol (n=294)	Sevoflurane (n=371)	Desflurane (n=393)	p-value
**Age (years)**	69.8 ± 6.3	69.2 ± 5.8	70.3 ± 6.5	69.7 ± 6.5	n.s.
**Gender male / female (%)**	54 % / 46%	34% / 66%	67 % / 33%	55.5% / 44.5%	<0.001
**BMI**	27.1 ± 5.1	27.2 ± 5.2	27.8 ± 5.4	26.4 ± 4.7	<0.001
**ASA status 1 / 2 / 3 / 4 (%)**	3.1% / 49.1% / 45.8% / 1.9%	3.4% / 54.4.% / 40.8 % / 1.4%	2.7% / 45.6% / 49.6% / 2.2%	3.3% / 48.6% / 46.1% / 2.0%	n.s.
**MMSE preoperative****	28.9 ± 1.4	29 ± 1.4	28.9 ± 1.4	28.9 ± 1.5	n.s.
**Midazolam Premedication yes / no (%)**	5% / 95%	4% / 96%	5% / 95%	6% / 94%	n.s.
**Surgery duration (min)**	171 ± 102	151 ± 89	162 ± 97	195 ± 110	0.000
**Surgical specialty (%)**					<0.001
**General surgery**	49,1%	20%	26%	54%	
**Orthopedics**	29.7%	31%	48%	21%	
**Urology**	8.1%	60%	11%	29%	
**Gynecology**	10.6%	34%	63%	3%	
**other**	2.5%	15%	23%	62%	
**Outcome parameter**					
**Discharge (%)**					0.021
**Home**	88.9%	92.8%	87%	87.8%	
**Other hospital or nursing home**	8.9%	6.9%	10.8%	8.6%	
**death**	2.2%	0.3%	2.2%	3.6%	

**Burst suppression duration (min)**	22.6 + 17.4	32.5 + 20.3	17.1 + 12.6	20.2 + 16.0	0.000
**Burst suppression ratio**	0.19 + 0.10	0.25 + 0.08	0.16 + 0.09	0.18 + 0.12	0.000
**Postoperative delirium NoPOD / POD (%)**	81% / 19%	87% / 13%	84% /16%	75% / 25%	0.000

Table S1: Patients' characteristics for all and for each one of the three anesthetic agent groups propofol, sevoflurane and desflurane. Differences calculated between the different anesthetic agents are seen in sex, were female patients more likely received a *total intravenous anesthesia with propofol*. Patients with *desflurane anesthesia* had a lower BMI, surgery duration was prolonged and patients underwent more frequently a general surgery, *sevoflurane anesthesia* was most frequently given in gynecological surgeries and *propofol anesthesia* was most frequently seen in urological surgery. We found no significant differences in age, ASA status, preoperative MMSE score and premedication with midazolam between the patients groups. Differences in age, BMI, MMSE, surgery duration were calculated by Kruskal-Wallis test, differences in sex, ASA status, MMSE score, surgery specialty, POD and discharge were calculated by chi-square test.

Correlations

			POD outcome	age (years)	ASA status	preoperative MMSE score	Surgery Speciality	surgery duration (min)	Anesthetic agent (propofol, sevoflurane, desflurane)	average BIS (mean intraoperative)	minimum BIS (mean intraoperative)	burst suppression ratio	burst suppression duration (min)
Spearman's rho	POD outcome	Correlation Coefficient	1,000	,178**	,140**	-,090**	-,125**	,279**	,133**	-,016	-,031	,006	,113**
		Sig. (2-tailed)	.	,000	,000	,004	,000	,000	,000	,657	,357	,839	,000
		N	1058	1058	1058	1001	1054	1058	1058	785	895	1058	1058
	age (years)	Correlation Coefficient	,178**	1,000	,143**	-,096**	-,033	-,023	,006	-,027	,014	,062*	,038
		Sig. (2-tailed)	,000	.	,000	,002	,282	,464	,857	,454	,681	,042	,222
		N	1058	1058	1058	1001	1054	1058	1058	785	895	1058	1058
	ASA status	Correlation Coefficient	,140**	,143**	1,000	-,127**	-,204**	,073*	,039	,050	,038	-,002	,042
		Sig. (2-tailed)	,000	,000	.	,000	,000	,017	,208	,160	,253	,935	,173
		N	1058	1058	1058	1001	1054	1058	1058	785	895	1058	1058
	preoperative MMSE score	Correlation Coefficient	-,090**	-,096**	-,127**	1,000	,033	-,004	-,045	-,010	,032	,000	,026
		Sig. (2-tailed)	,004	,002	,000	.	,300	,906	,157	,796	,356	,989	,403
		N	1001	1001	1001	1001	997	1001	1001	740	846	1001	1001
	Surgery Speciality	Correlation Coefficient	-,125**	-,033	-,204**	,033	1,000	-,131**	-,307**	-,024	-,017	,070*	,066*
		Sig. (2-tailed)	,000	,282	,000	,300	.	,000	,000	,499	,605	,023	,033
		N	1054	1054	1054	997	1054	1054	1054	783	891	1054	1054
	surgery duration (min)	Correlation Coefficient	,279**	-,023	,073*	-,004	-,131**	1,000	,179**	-,028	-,020	-,193**	,283**
		Sig. (2-tailed)	,000	,464	,017	,906	,000	.	,000	,433	,558	,000	,000
		N	1058	1058	1058	1001	1054	1058	1058	785	895	1058	1058
	Anesthetic agent (propofol, sevoflurane, desflurane)	Correlation Coefficient	,133**	,006	,039	-,045	-,307**	,179**	1,000	,029	-,006	-,306**	-,253**
		Sig. (2-tailed)	,000	,857	,208	,157	,000	,000	.	,413	,850	,000	,000
		N	1058	1058	1058	1001	1054	1058	1058	785	895	1058	1058
	average BIS (mean intraoperative)	Correlation Coefficient	-,016	-,027	,050	-,010	-,024	-,028	,029	1,000	,990**	,054	,033
		Sig. (2-tailed)	,657	,454	,160	,796	,499	,433	,413	.	,000	,129	,357
		N	785	785	785	740	783	785	785	785	785	785	785
	minimum BIS (mean intraoperative)	Correlation Coefficient	-,031	,014	,038	,032	-,017	-,020	-,006	,990**	1,000	,051	,033
		Sig. (2-tailed)	,357	,681	,253	,356	,605	,558	,850	,000	.	,129	,330
		N	895	895	895	846	891	895	895	785	895	895	895
	burst suppression ratio	Correlation Coefficient	,006	,062*	-,002	,000	,070*	-,193**	-,306**	,054	,051	1,000	,555**
		Sig. (2-tailed)	,839	,042	,935	,989	,023	,000	,000	,129	,129	.	,000
		N	1058	1058	1058	1001	1054	1058	1058	785	895	1058	1058
	burst suppression duration (min)	Correlation Coefficient	,113**	,038	,042	,026	,066*	,283**	-,253**	,033	,033	,555**	1,000
		Sig. (2-tailed)	,000	,222	,173	,403	,033	,000	,000	,357	,330	,000	.
		N	1058	1058	1058	1001	1054	1058	1058	785	895	1058	1058

**. Correlation is significant at the 0.01 level (2-tailed).

*. Correlation is significant at the 0.05 level (2-tailed).

Table S2: Spearman Rho correlation for POD and different known risk factors. We found a high correlation of POD with age, ASA status, MMSE score, surgery duration, surgery specialization, burst suppression duration and anesthetic agent given for maintenance, but no correlation for average BIS level, minimum BIS level and burst suppression ratio.

	**All Patients (n=1,058)**			**Propofol (n=294)**			**Sevoflurane (n=371)**			**Desflurane (n=393)**		
	**No-POD (n=860)**	**POD (n=198)**	**p-value**	**No-POD (n=256)**	**POD (n=38)**	**p-value**	**No-POD (n=311)**	**POD (n=60)**	**p-value**	**No-POD (n=293)**	**POD (n=100)**	**p-value**
**Burst suppression duration (min)**	21.4 ± 16.2	27.5 ± 21.3	0.000	30.8 ± 18.9	44.1 ± 25.0	0.000	15.8 ± 11.5	23.9 ± 15.4	0.000	19.1 ± 14.2	23.5 ± 20.0	0.025
**Burst suppression ratio**	0.19 ± 0.1	0.2 ± 0.11	n.s.	0.24 ± 0.08	0.26 ± 0.1	n.s.	0.16 ± 0.09	0.17 ± 0.06	n.s.	0.18 ± 0.11	0.19 ± 0.12	n.s.
**Surgery duration**	157 ± 92	175 ± 127	0.000	143 ± 83	205 ± 106	0.000	153 ± 90	208 ± 117	0.000	172 ± 98	264 ± 116	0.000

Table S3: Burst Suppression parameters for the single anesthetic agent groups for NoPOD and POD patients.
